# A competency based educational programme for research nurses: an Italian experience

**DOI:** 10.3332/ecancer.2009.134

**Published:** 2009-03-18

**Authors:** S Liptrott, L Orlando, M Clerici, A Cocquio, G Martinelli

**Affiliations:** 1 Division of Haemato-oncology, European Institute of Oncology, Milan, Italy; 2 Supportive Therapy and Palliative Care Unit, European Institute of Oncology, Milan, Italy

## Abstract

**Background:**

the EU Directive states the requirement of staff working in trials to be qualified by education, training and experience [14]. This includes the research nurse; however, in the transition from ward nurse to research nurse, new and highly developed skills and knowledge are required in order to work effectively.

**Methods:**

an educational programme was developed, which included a review of current knowledge and baseline practice, development of competencies related to the role of research nurse, haemato-oncology and clinical trial education to support this advanced practice for nurses in clinical trials.

**Results:**

overall, the feedback on the course by the nurses was very positive, and the nurses were able to undertake the role of research nurse within specified clinical trials.

## Introduction

The treatment options for cancer patients are both varied and complex, and many patients are offered inclusion into clinical trials or research programmes where effective treatments are promoted and supported [1]. Safe and appropriate performance of clinical trials is necessary to the advancement of treatment in a scientifically sound manner [2], the performance of which regularly incorporates the figure of the research nurse within the team. Many authors have reported the value of the role in enhancing the safety and effectiveness of conducting clinical trials [3–6] by providing a holistic approach to care through the process of assessment, planning, implementation and evaluation.

The research nurse has been described as a vital link between the patient, principal investigator, study sponsor and administrative staff, demonstrated by increased number of studies undertaken, patient recruitment and completion rates following employment of appropriately skilled nurses [7], reduction of deficits or incomplete data [1] and positive outcomes for patients in nurse-managed trial conclusion [8].

Within this role, nurses must be equipped to function at a higher level, reflecting their higher degree of autonomy and involvement in direct decision-making regarding patient care [9,10]. One significant work by Ehrenberger and Lilington [11] in the development of a tool to delineate the role of the research nurse, identified key themes (Table 1) following an extensive literature review, item generation and expert review. Within these themes, the authors go on to elaborate further aspects of each section/theme. This tool provides a significant basis in beginning to understand what variation exists in the role of the research nurse, and what the potential requirements of the role can be.

In order to equip nurses for the research nurse’s role, it is important to recognize their needs, provide support and provide adequate preparation both theoretical and practical in order to reflect what may be a new but is certainly an advanced sphere of nursing practice.

This paper describes a project of educational development and preparation of two experienced Italian nurses to work as research nurses within the haemato-oncology unit of a leading research hospital in Italy.

## Background

In Italy during 2006, approximately 250,000 new cancer diagnoses were made [12]. There were 1389 oncology clinical trials (not including phase I trials) registered with the Italian national monitoring centre for clinical trials, Osservatorio Nazionale Sperimentazione Clincia, between January 2000 and December 2007 [13], around 27.8% of total clinical trial studies in Italy.

The EU Directive 2005/28/EC states the requirement of staff working within trials to be qualified by education, training and experience [14]. Although the role of the research nurse is not directly named within the directive, they have an active role and are therefore required to comply with this. However in the transition from ward nurse to research nurse, new and highly developed skills and knowledge are required in order to work effectively. The complexity of oncology clinical trials requires nurses to have knowledge of medical systems, implications of study treatment and problem solving skills to deal with unforeseen events for which the nurse can provide assistance [1]. Stevens and Hill [15] suggest the provision of research nurse education and training is very much dependant in the local infrastructure and support, and is not consistent. There are often no formal educational structures for the acquisition of these new skills [10,16,17]. This experience of a disparity in structured training for the research nurse is mirrored within Italy.

Support is required for healthcare professionals to develop knowledge and skills required to work in such a challenging and rapidly advancing field. In an effort to support two nurses starting within the post of research nurse, an educational programme was devised.

## Objectives

The overall aim of this project was to provide a programme of education and support for the development and integration of two nurses into the role of research nurse. More specific objectives were:

- To provide a broad yet detailed education base for nurses working within the management of haemato-oncology patients in clinical trials through the implementation of a competency based education programme
- To demonstrate the efficacy of the nurses in the role of the research nurse in terms of functional integration into an existing research team.
- To evaluate the efficacy of the education programme through demonstration of skill development and participant evaluation

## Developing the educational programme

### Introducing the role of the research nurse

Although various themes in the role of the research nurse are identified in the literature, it was important to recognize that this would be a new figure with the division. Support from managers as well as medical and nursing staff was vital to the success of the project, including the release of the staff to undertake the course and practical support in the clinical setting.

Some medical staff had experience of working with a research nurse in other hospitals, but it was decided to provide a short presentation on the role of the research nurse to all staff in the department, demonstrating how this figure could complement and improve standards of care for patients within clinical trials.

Following this presentation a plan of action was defined by myself as course co-ordinator, the lead clinician and the lead nurse in relation to the course. Firstly, a review of the current knowledge and baseline practice of the nurses undertaking the course was performed. Secondly, the course programme would be developed in line with the educational needs of the nurses, and finally, an evaluation of the course would be undertaken by nurses and staff working within clinical trials where the nurses had become an integral part.

### Reviewing current knowledge and baseline practice

A baseline evaluation of the nurses’ knowledge and skills highlighted interesting findings. The nurses undertaking the educational programme had practical experience working within the department for between five and eight years, they were actively undertaking nursing research within the field of haemato-oncology and stem cell transplant. Both nurses were already qualified to degree level and were regularly participating in educational updates in accordance with institutional and regulatory guidelines, and personal interest.

Although the regulation of nursing education started in 1925 in Italy with guidance for ‘generalist nurses’, more specialist training arrived much later for nurses who wish to achieve a professional level necessary to perform specific tasks in the fields of advanced clinical and healthcare practice, management, education and research [18]. They are focused primarily in fields of co-ordinator (for ward management posts), critical care, paediatrics and midwifery, elderly care and psychiatry. There are only a few post-registration courses for care of terminal patients, palliative cancer and oncology or research [19], and it is unclear if there is anything specific for the field of haemato-oncology / haematopoietic stem cell transplantation.

The Deontological Code of Nursing in Italy sets out the ethical parameters according to which those belonging to the profession must work [20]. Article 3 refers to nursing actions for which education and updating are necessary. Emphasis is given to the link between knowledge and responsibility ‘nurses take their responsibility according to the level of knowledge achieved’. In order to support the nurses in this advanced practice role, it was necessary to provide both support and education in order to develop their knowledge base and assume the role and responsibilities of the research nurse. The Oncology Nursing Society [21] states that ‘coordination of clinical trials is best accomplished by nurses who have been educated and certified in oncology nursing’ (p189). A disease-related post-registration qualification can be utilized in part to provide a baseline for the research nurse, where an understanding of patho-physiology and related treatments is important as a foundation for providing support and care for their patient group. Despite years of clinical experience in haemato-oncology, the nurses did not have a specific oncology related academic course. It was therefore recognized that although an academic qualification could not be provided, this aspect of a revision of haemato-oncology patient care based on evidence and research, should be incorporated into the course programme.

### Development of the educational programme

In order to demonstrate the preparation and development of their practice, competencies related to the role were developed to provide legitimate and realistic goals for nurses to achieve. Competency based learning is a method of education that allows for flexibility, reduction of duplicity, and building on previous knowledge [22,23].

Literature suggests that competency can be established based on how well individuals integrate knowledge or skills in performing functions, duties or tasks [24,25]. This integration into practice and use of critical analytical thinking, is a vital component for the research nurse dealing with complex issues and problems in clinical trials. Girot [26] suggested professional education should be focused in the development of critical analytical skills, which help to prepare them to respond more readily to change. By including knowledge and understanding in the performance of work tasks, the intention was to provide a more holistic approach to nursing care.

Competencies have been developed as a way of the setting standards of what is the baseline acceptable level of competence and to help identify nursing as a professional occupation [27,28]. This can be useful in demonstrating standards where none currently exist within the hospital.

Development of clinically based competencies with respect to clinical trials included consideration of the following aspects:

1. legislation in relation to clinical trial performance in Italy—including relevant international, European and national legislation
2. national legislation in relation to clinical practice guidance for nurses
3. local hospital guidelines—both in clinical trial performance and nursing practice
4. review of existing competency programmes in this field
5. roles of the research nurse identified by literature review, job analyses and interviews with research nurses

### Development of a ‘checklist’ of competencies

A checklist of key competencies of the research nurse role was identified. These were aimed at areas that reflected the needs of the nurses and the environment in which they would be working. These competencies were designed to cover potential aspects of the research nurse’s role in the clinical trial pathway, from the planning phase of clinical trial development, knowledge and impact of guidance for trials (local, national and international), protocol writing, feasibility assessments, multidisciplinary team working, problem solving skills, day-to-day running of trials, management of serious and non-serious adverse events, through to clinical trial planned closure, research interpretation and result writing.

The competencies were written in terms of a broad statement of what the nurse would be able to do, but then broken down into more detailed list of criteria, which reflected the policies and procedures locally for clinical trial development and management, and the disease speciality. The competencies were reviewed by the nurse manager and lead consultant working within clinical trials. Final modifications were made based on their suggestions and the competencies were given to the nurses undertaking the educational programme.

The educational programme itself was delivered by the course co-ordinator working with the two nurses. For these nurses, working specifically in the field of haemato-oncology, however, it was agreed that firstly providing an evidence-based theoretical background to the area of care in haematology-oncology, including nursing care aspects (Table 2) would be an appropriate way to update their existing knowledge. The content of the haemato-oncology sessions was based on the EONS Post Basic Curriculum in Cancer Nursing [29]. Following this, the educational programme focused on clinical trials with lectures on various aspects including those where the research nurse is directly involved (Table 2).

These sessions were supported by other activities included self-directed study, critiques of clinical cases, clinical trials, research papers, interpretation of results and application of findings etc, case studies on issues including informed consent, legal and ethical aspects of clinical trials, including informatics (Table 3). The initial teaching phase took place over six weeks. After this period, the nurses were supervised by the course co-ordinator in undertaking clinical and practical experience in the phases of design, planning and implementation and day-to-day running of protocols.

### Competency assessment

Competency assessment is also crucial to the identification of areas for professional development and educational needs [30]. Methods used to assess achievement of competencies included observation, supervisory assessments, ability and knowledge tests, portfolios and self-assessment. While there are limitations of a self assessment approach, such as subjectivity and response bias, several authors suggest that self-assessments, if carried out correctly, can form an important part of a comprehensive assessment [31,32].

McMullan *et al* [33] and Williams [34] suggest that the process of developing a portfolio may help students in acquiring such skills, as the students are responsible for creating the portfolios, and are taking responsibility for their own learning and development. The theoretical basis of the portfolio approach in this framework is underpinned by the four assumptions of the theory of adult learning suggested by Knowles [22]: (i) the student is self-directed; (ii) the student’s past experiences are a rich resource for learning; (iii) readiness to learn develops from life tasks and problems; and (iv) the student demonstrates curiosity and is self-motivated to grow and achieve. Even if everyone does not have these tendencies, portfolio preparation can help to nurture and develop them, given a facilitative climate [35].

Using this theory, the nurses were asked to produce a portfolio of evidence to support their professional development. This portfolio of evidence was a new concept for the nurses as this is not standard practice in Italy. The format of the portfolio was therefore directed by the course co-ordinator and not the nurses, and was centred around the competencies with the incorporation of reflective pieces. The main body of the portfolio comprised of evidence of knowledge and the application of knowledge in practice. The portfolio allows for evaluation of competencies that may otherwise be difficult to assess, such as practice-based improvements, use of scientific evidence in practice, professional behaviour, and creative endeavours [36].

The nurses were successfully integrated into the role of research nurse within the department, with the responsibility for following specific identified clinical trials. Overall, the feedback on the course by the nurses was very positive, acknowledging: ‘***topics were important for my professional growth’*** and having ‘***stimulated an improvement within my professional role’***.

The project was evaluated by the participants and members of the research team. The nurses participating in the programme were asked to complete a questionnaire where they identified that the topics covered in the ‘general’ haemato-oncology sessions were relevant but they wanted further information regarding genetics, pharmacogenomics and stem cell transplantation. With regards to the clinical trial/research training, the topics were relevant but both nurses identified the need for further detail regarding statistical analysis. Both nurses reported that the self-directed study set was relevant and well supported and there was time available for its completion; however, overall there were not enough sessions.

The nurses were also able to demonstrate attainment of the competencies by the use of the practice portfolio. Although this was a new concept for the nurses, they said it was useful in both personal recognition of all the work they had done, and demonstrating development of skills and competencies to others who were not familiar with the role of research nurse. The reflective pieces were suggested by the nurses to be useful in identifying strengths, weaknesses and areas for development, similar to findings in the literature [37,38]. Portfolios were written in a combination of English and Italian, to facilitate completion. They were evaluated with the written competencies by the course co-ordinator and the Divisional Director. The amount of evidence to be provided and the time-consuming aspect of compiling the portfolio may have a negative effect on the student’s motivation [39] although the nurses compiling these portfolios felt it was useful to be able to demonstrate their development and the work achieved.

## Discussion

The aim of this project was to provide an educational programme and support to nurses in their new role of research nurse within the department. Results were positive in terms of demonstration of competency in clinical practice and feedback by the nurses regarding the utility of the programme. It was clear from the review that there were many and varied aspects to the research nurse role, not all of which could be incorporated in-depth in a short, intensive training programme. The programme aimed to reflect the needs of both the nurses in consolidating their experience and developing skills to perform the role of the research nurse within a specified area.

Baseline knowledge and learning was difficult to assess in terms of documented course attendance and access to such courses. The nursing educational structure difference between Italy and the UK were notable. This was not to be ignored in review of baseline practice and knowledge outside of formalized courses.

This was a competency based course incorporating competencies and research based findings from existing literature. It is recognized that this is not a ‘standard’ tool for research nurse education, as there are existing competencies, yet analysis showed that these did not fully reflect the educational needs of these particular nurses, and as such further areas were supplemented to support their practice development. It should be highlighted, though, that the core content of the educational programme reflected the items generated in the Clinical Trials Nurse Questionnaire by Ehrenberger and Lillington [11].

Methods suggested for the evidence of competencies, encouraged the demonstration of critical thinking and analysis, both of patient care issues and protocol related issues. Some methods, such as case presentations and trial protocol critical analysis, were novel approaches for the nurses who reported that the experience was rewarding and an allowed for an acknowledgement of their already extensive clinical experience. This was an intensive educational programme, with the course co-ordinator dedicated to this project. Although this may not be possible in all settings, with a core of research nurses, they could with appropriate support provide a mentoring figure for new research nurses.

The methods for assessment of whether the competencies were achieved was not formally tested for validity or reliability, only between professionals within the department of haemato-oncology. Evaluation of educational programmes appears to be an inherent problem [40]. More rigorous methods should be incorporated, perhaps using examples from other areas such as those developed in line with cancer educational programmes [41], however, this type of method with statistical analysis may be of limited use if the number of staff undertaking the programme is small.

## Conclusions and implications for practice

This paper has outlined the development of an educational programme for the preparation of two nurses to undertake the role of research nurse. The ultimate aim of producing competent nurses is to guarantee that patients or clients receive a high standard of care [42], a vital aspect in clinical trial performance. The role of the research nurse is complex and varied according to the requirements of the research they are involved with. First level nurse training does not cover all aspects and skills required to perform this role effectively, however by assessing the clinical practice environment and requirements of nurses working in clinical trials, it was possible to recognize the previous education, skills and knowledge of the nurses and develop a programme to complement this. The development of the job specific training and competencies have been helpful in clarifying and defining key areas of a previously poorly defined role, and have assisted these nurses in performing as research nurses within in clinical trials.

Further recommendations of this study are:

- development of a standardized format for research nurse competencies, that could be tested for validity and reliability in its application in other departments
- to provide education and training that suits the work of the research nurses —e.g. core generic aspects and those more specific, such as disease-related, phase-related, chemo-prevention, etc
- identify the ongoing training and education needs of the research nurses through the forum of appraisals and development of personal objectives.

## Figures and Tables

**Table 1:**
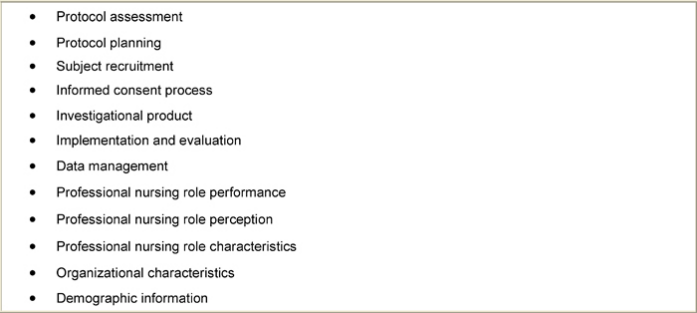
Sections identified in the Clinical Trials Nursing Questionnaire®© [11]

**Table 2:**
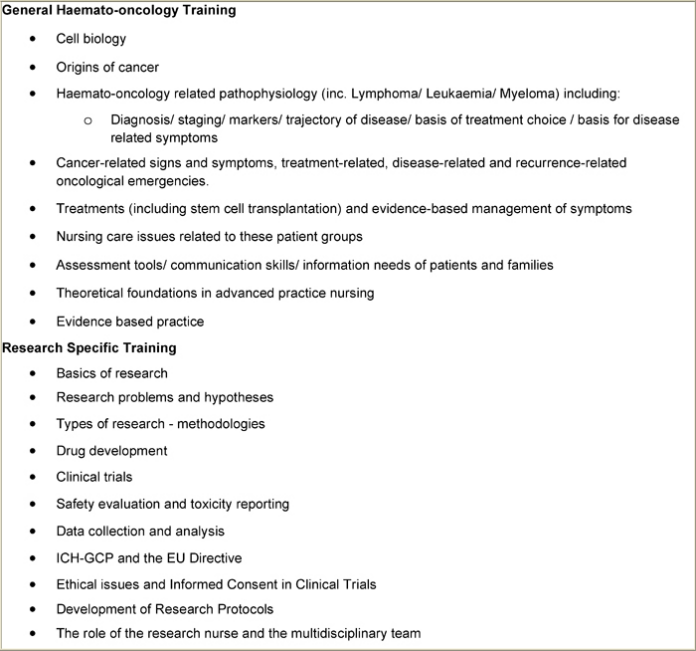
Educational sessions/discussion in the Research Nurse Education Programme

**Table 3:**
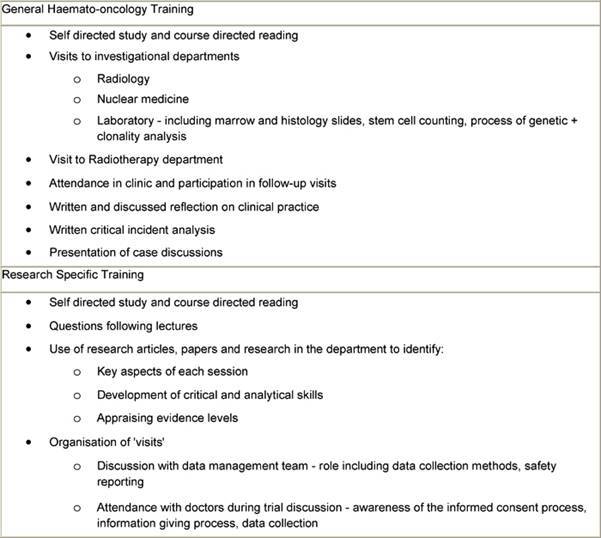
Methods to support educational sessions
